# STING1 in Different Organelles: Location Dictates Function

**DOI:** 10.3389/fimmu.2022.842489

**Published:** 2022-03-17

**Authors:** Ruoxi Zhang, Rui Kang, Daolin Tang

**Affiliations:** Department of Surgery, University of Texas (UT) Southwestern Medical Center, Dallas, TX, United States

**Keywords:** adaptor protein, autophagy, cell death, immunity, organelle, STING1

## Abstract

Stimulator of interferon response cGAMP interactor 1 (STING1), also known as TMEM173, is an immune adaptor protein that governs signal crosstalk that is implicated in many physiological and pathological processes. Although it has been established that STING1 traffics from the endoplasmic reticulum (ER) to Golgi apparatus (Golgi) upon DNA-triggered activation, emerging evidence reveals that STING1 can be transported to different organelles, which dictate its immune-dependent (e.g., the production of type I interferons and pro-inflammatory cytokines) and -independent (e.g., the activation of autophagy and cell death) functions. In this brief review, we outline the roles of STING1 in different organelles (including the ER, ER-Golgi intermediate compartment, Golgi, mitochondria, endosomes, lysosomes, and nucleus) and discuss the potential relevance of these roles to diseases and pharmacological interventions.

## Introduction

Stimulator of interferon response cGAMP interactor 1 (STING1, also known as STING or TMEM173), an evolutionarily conserved transmembrane protein, is expressed in various endothelial and epithelial cell types as well as in T cells, B cells, and myeloid cells (e.g., macrophages and monocytes) and mainly localized on endoplasmic reticulum (ER). It was originally described as an adaptor protein that mediated the production of type I interferons (IFNs) in DNA-induced immune responses (1–4). Although STING1-mediated innate immunity plays significant roles in shaping host defense against microbe invasion and tumor growth (5), aberrative activation of STING1 can also disturb immune balance, thereby leading to pathological conditions and human diseases, such as STING-associated vasculopathy with onset in infancy (SAVI), Aicardi-Goutieres syndrome, systemic lupus erythematosus (SLE), and sepsis (6, 7), as well as neurodegenerative diseases (8), and metabolic diseases (9). Therefore, STING1 is an emerging therapeutic target in translational research.

Accumulating evidence demonstrates that the subcellular distribution of STING1 is not restricted to an ER-to-Golgi apparatus (Golgi) membranous network. Under different circumstances, the localization on other organelles enables some immune-independent functions of STING1, contributing to autophagy (10), regulated cell death (11), ER stress (12), lipid metabolism (13), and DNA damage response (DDR) (14). The participation and crosstalk of these organelles determine the function of STING1 in diseases by controlling its location, binding partners, and signaling recognition. In this mini-review, we highlight recent scientific advances regarding STING1 in different subcellular structures, and will draw parallels and differences across functions and diseases.

## Roles of STING1 in Organelles

### ER

**Figure 1:**
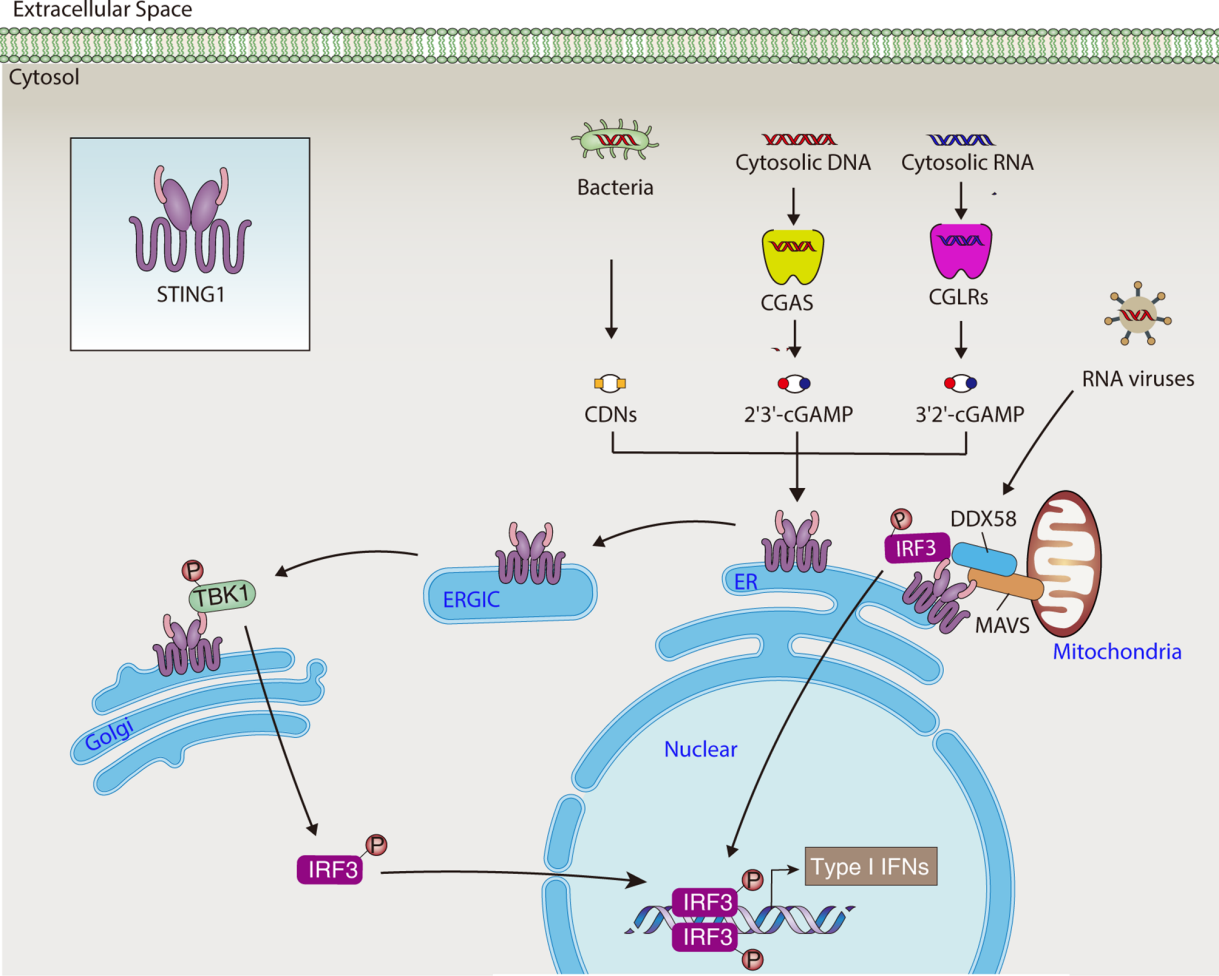
STING1-mediated type I IFNs production. In response to DNA or RNA from pathogens and hosts, STING1 activates the TBK1-IRF3 pathway, leading to the production of type I IFNs. CDN, cytosolic cyclic dinucleotide; cGAMP, cyclic GMP-AMP; CGAS, cyclic GMP-AMP synthase; CGLR, CGAS-like receptor; DDX58, DExD/H-box helicase 58; ER, endoplasmic reticulum; ERGIC, endoplasmic reticulum-Golgi intermediate compartment; IRF3, interferon regulatory factor 3; MAVS, mitochondrial antiviral signaling protein; STING1, Stimulator of interferon response cGAMP interactor 1; TBK1, TANK binding kinase 1; type I IFNs, type I interferons.

STING1 possesses an N-terminal transmembrane (TM) domain that spans the ER membrane four times, a cytosolic cyclic dinucleotide (CDN) binding domain (CBD), and a C-terminal tail (CTT) (15). Under steady-state conditions, STING1 is retained in the ER by its ER-binding partners, with its CBD face to the cytosol, which facilitates the detection of second messengers. The second messenger is the small molecule and ion that transmits the signal received by the cell surface receptor to the effector protein. The cytosolic DNA sensor cyclic GMP-AMP synthase (CGAS) recognizes various DNA driven from pathogens or hosts, then generates the second messenger’s cyclic guanosine monophosphate-adenosine monophosphate (cGAMP). The cGAMP or bacteria-produced CDNs bind to STING1, causing STING1 to activate through conformational changes and oligomerization. This protein secondary structural change of STING1 subsequently promotes the translocation of STING1 from ER to Golgi, where the CTT of STING1 binds and phosphorylates the TANK binding kinase 1 (TBK1) and interferon regulatory factor 3 (IRF3). Finally, activated IRF3 promotes gene transcription of type I IFNs (**Figure 1**). Alternatively, STING1-mediated nuclear factor kappa B (NF-κB) pathway activation favors the production of proinflammatory cytokines, such as tumor necrosis factor (TNF) and interleukin 6 (IL6). This supports the hypothesis that STING1 is a mediator of inflammatory disease (16).

Although there is no clear consensus on the ER targeting signal sequence of STING1, several binding partners of STING1 regulate the ER retention of STING1. The ER calcium sensor, stromal interaction molecule 1 (STIM1), physically interacts with and retains STING1 in ER, inhibiting downstream immune responses and ER stress in mouse embryonic fibroblasts (MEFs) and human embryonic kidney 293 (HEK293) cells (17). In contrast, transmembrane protein 203 (TMEM203) competes with STIM1 to bind STING1, promoting cGAMP-induced STING1 activation in human macrophages (18). In addition, toll interacting protein (TOLLIP), an ubiquitin-binding protein that interacts with several components of the toll-like receptor, can stabilize STING1 on the ER and reduce cGAMP-induced lysosomal degradation of STING1 in MEFs (19). These findings reinforce the notion that STING1 is an adaptor protein with strong activity in binding multiple proteins. Different STING1 protein complexes not only affect the location of STING1 in the ER, but also regulate its degradation and activity.

**Figure 2:**
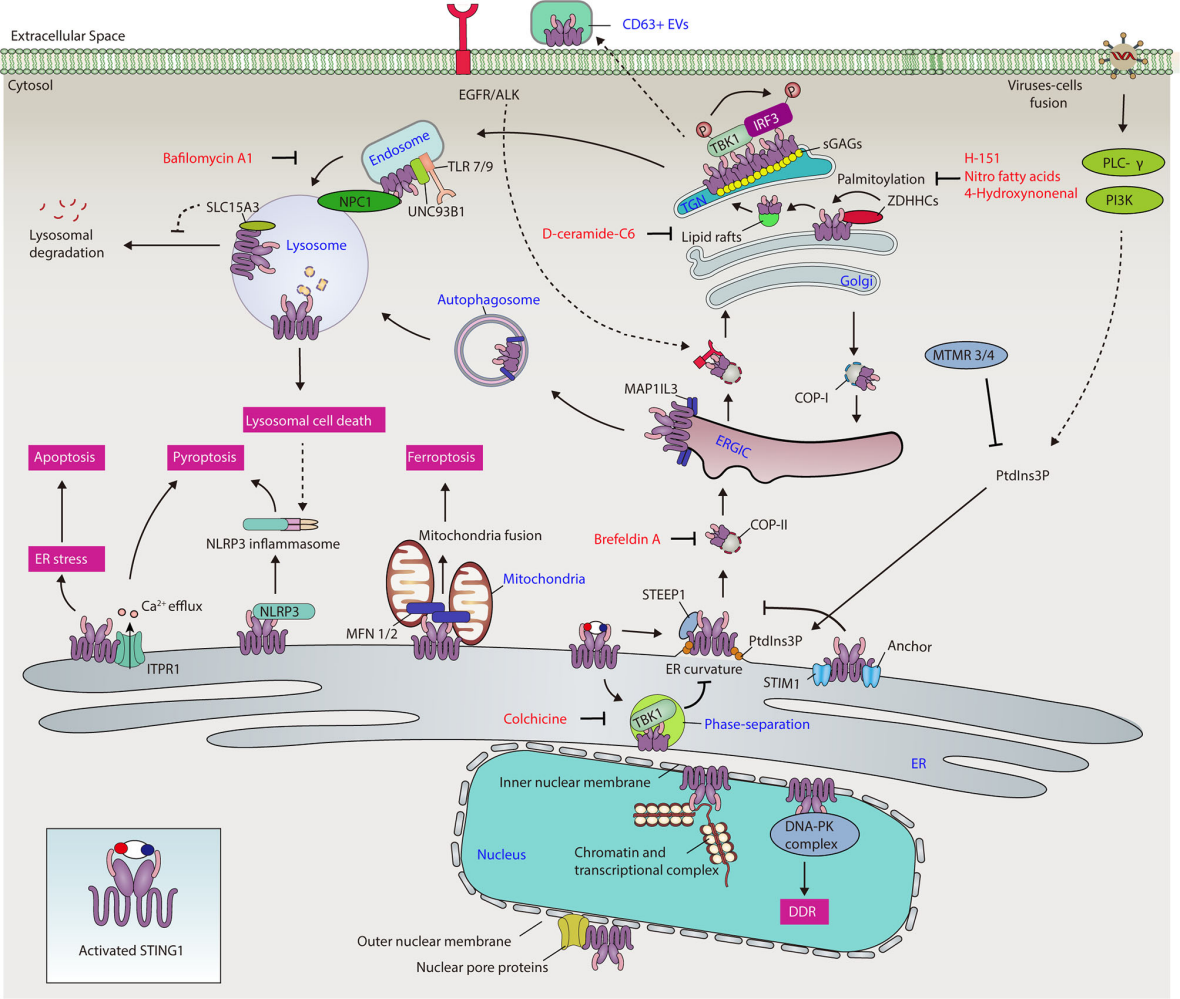
The network of STING1 in different organelles. Activated STING1 traffics to the ER, ERGIC, Golgi, mitochondria, endosomes, lysosomes, and nucleus, contributing to different cellular processes, including immune response, ER stress, cell death, autophagy, gene transcription, and DNA damage response. ALK, ALK receptor tyrosine kinase; Ca^2+^, calcium; COP-I, coat protein complex I; COP-II, coat protein complex II; DDR, DNA damage response; DNA-PK, DNA-dependent protein kinase; EGFR, epidermal growth factor receptor; ER, endoplasmic reticulum; ERGIC, endoplasmic reticulum-Golgi intermediate compartment; EV, extracellular vesicle; IRF3, interferon regulatory factor 3; ITPR1, inositol 1,4,5-trisphosphate receptor type 1; MAP1LC3, microtubule-associated protein 1 light chain 3; MFN 1/2, mitofusin 1/2; MTMR3/4, myotubularin-related protein 3/4; NLRP3, NLR family pyrin domain containing 3; NPC1, Niemann-Pick type C1; PI3K, phosphatidylinositol-3 kinase; PLC-γ, phospholipase C-γ; PtdIns3P, phosphatidylinositol-3-phosphate; sGAG, sulfated glycosaminoglycan; SLC15A3, solute carrier family 15 member 3; STING1, Stimulator of interferon response CGAMP interactor 1; STIM1, stromal interaction molecule 1; STEEP1, STING1 ER exit protein 1; TBK1, TANK binding kinase 1; TGN, trans-Golgi network; TLR, toll-like receptor; type I IFN, type I interferon; UNC93B1, unc-93 homolog B1, TLR signaling regulator; ZDHHC, zinc finger dhhc-type palmitoyltransferase.

The exit of STING1 from the ER is a kinetic process involving interaction with STING1 ER exit protein 1 (STEEP1) to recruit phosphatidylinositol 3-kinase catalytic subunit type 3 (PIK3C3), leading to phosphatidylinositol-3-phosphate (PtdIns3P) synthesis and membrane curvature on the ER, and finally packaging STING1 into vesicles that bud from the ER (20) (**Figure 2**). STING1 mutations in the TM domain cause constitutive ER exit and subsequent autoimmunity in SAVI patients, the process of which is independent of cGAMP binding. Brefeldin A, an inhibitor of protein trafficking between the ER and Golgi, blocks STING1 trafficking regardless of its mutation status (21). These data indicate that the membrane topology of STING1 is attributed to its transmembrane domain and PtdIns3P synthesis. To support this, virus-mediated cell fusion, including that of DNA viruses (e.g., herpes simplex virus type 1 [HSV-1]) and RNA viruses (e.g., influenza A virus), can activate membrane-proximal signaling phospholipase C-γ (PLC-γ) and phosphatidylinositol-3 kinase (PI3K) pathways, triggering STING1-dependent immune responses independent of nucleotide recognition (22, 23). Overall, these data suggest that PtdIns3P acts as a key signal to promote ER exit of STING1. Given that STING1 has two calcium (Ca^2+^) binding sites and PtdIns3P triggers Ca^2+^ efflux from ER to induce STING1 activation (24, 25), Ca^2+^ may be another ER exit signal for STING1.

Phase separation, the process of spontaneous separation of a mixed solution of macromolecules (such as a protein or nucleic acid) into two phases, is an alternative location for STING1 after ER exit. Functionally, to prevent STING1 overactivation, a high cGAMP level induces STING1 condensation on the ER and the formation of a puzzle-like droplet that separates STING1 and TBK1 from downstream IRF3 (26) (**Figure 2**). However, the constitutive activating mutants in STING1 (including N154S or V155M mutations) reduce STING1 phase separators. The lack of this sponge-based negative feedback mechanism contributes to the autoimmunity of patients with SAVI. The microtubule inhibitor colchicine (but not actin polymerization inhibitors or brefeldin A) inhibits STING1 condensation, indicating that the ER exit of STING1 is also microtubule-dependent.

Many recent studies have focused on how ER-associated STING1 modulates ER stress and cell death (**Figure 2**). For example, during HSV-1 infection, STING1 inhibits the ubiquitin degradation of NLR family pyrin domain 3 (NLRP3) in macrophages through direct protein-protein interaction in the ER, causing inflammasome activation and subsequent pyroptosis (27). In bacterial sepsis, the interaction of STING1 with ER Ca^2+^ channel inositol 1,4,5-trisphosphate receptor type 1 (ITPR1) increases Ca^2+^ release from ER to cytoplasm in macrophages, leading to GSDMD-dependent pyroptosis, the release of coagulation factor III, and subsequent activation of systemic coagulation in septic mice (25, 28). The constitutive activation of STING1 can also trigger apoptosis in CD8^+^ T cells by disrupting Ca^2+^ homeostasis and activating the unfolded protein response and ER stress through its CBD (12). In contrast, the interaction of notch intracellular signaling domain (NICD) with STING1 CBD limits STING1-mediated apoptosis in CD4^+^ T cells, preventing immunosuppression in the late stage of sepsis (29). Together, these findings establish STING1-dependent Ca^2+^ signaling in the control of regulated cell death in immune cells.

ER STING1 also links the lipid metabolism and immune response through the insulin signaling pathway (30–33). On one hand, STING1-mediated type I IFN signaling limits cholesterol biosynthetic flux in MEFs. On the other hand, insulin-induced gene 1 (INSIG1), sterol regulatory element binding transcription factor 1 (SREBP), and SREBF chaperone (SCAP) show strong activity to bind STING1, enhancing STING1-mediated cytokine production. These results raise the concern of whether lifestyle factors (such as a high-fat diet) affect STING1 signaling and thereby affect the outcome of metabolic diseases.

### ER-Golgi Intermediate Compartment

The ER-Golgi intermediate compartment (ERGIC) is a pre-Golgi intermediate composed of vesicular tubular clusters that deliver secretory cargo from ER exit sites to the Golgi (34). Upon activation, STING1 exits from ER and relocates to the Golgi *via* the ERGIC in a coat protein complex II (COP-II)-dependent manner (10, 35). Pharmacological or genetic inhibition of ER-to-ERGIC trafficking significantly suppresses STING1 activation (21, 36). On the other hand, coat protein complex I (COP-I) mediates the retrograde transport of STING1 to the ERGIC from Golgi, while deficiency in COP-I transport causes failure of Golgi-to-ER STING1 retrieval and ligand-independent activation of STING1, thus contributing to COPA syndrome (37–40) (**Figure 2**). COPA syndrome is a rare inherited autoimmune disease caused by mutations in the coat protein complex subunit alpha (COPA) gene. Studies highlight the importance of the ER-ERGIC/Golgi axis in the control of STING1 activation, demonstrate a “tug-of-war” between the ER and the ERGIC/Golgi for STING1, and suggest therapeutic strategies for inflammatory and autoimmune diseases (37–40). However, whether STING1 can be directly activated on ERGIC needs to be further clarified by inhibition of “ERGIC-to-Golgi” membrane trafficking.

In addition to its role in immune response, ERGIC is also involved in STING1-dependent autophagy, a process that relies on the formation of various membrane structures to degrade cytoplasmic cargo. ERGIC could serve as a membrane source for the formation of autophagosomes through STING1 activation-induced microtubule-associated protein 1 light chain 3 (MAP1LC3) lipidation. This STING1-mediated autophagy initiation is further enhanced by WD repeat domain, phosphoinositide interacting 2 (WIPI2) (10) or autophagy-related 16-like 1 (ATG16L1) (41). Given that STING1 directly interacts with MAP1LC3 upon activation, MAP1LC3 lipidation-mediated transportation may provide more trafficking routes for STING1 on intracellular organelles. Nevertheless, autophagy-mediated STING1 degradation limits the immune activity of STING1.

### Golgi

The Golgi connects the ER, mitochondria, endosomes, and other organelles through membranous networks for vesicular trafficking and protein/lipid secretion. It also serves as a platform for signaling transduction connecting multiple innate immune pathways (42). Consistently, activated and polymerized STING1 traffics to the Golgi and recruits TBK1 for the phosphorylation of IRF3, inducing a set of downstream immune and non-immune events, such as IRF3-mediated type I IFN production, apoptosis, and necroptosis (11). As a part of Golgi membranous networks, activated Golgi STING1 is further transported to endosomes for degradation or transported to ER for recycling (43).

The Golgi membranous network contains a tubular reticular network of membranes facing the ER, namely the cis-Golgi network (CGN, including ERGIC) and another tubular reticular network of membranes facing plasma membrane and compartments of the endocytic pathway, called the trans-Golgi network (TGN) (44). The regulation of ER-to-Golgi trafficking of STING1 is context-dependent. For example, phosphatidylinositol 3 phosphatases myotubularin-related protein 3 (MTMR3) and myotubularin-related protein 4 (MTMR4), which inhibits PtdIns3P production, thereby suppressing STING1 trafficking to Golgi and subsequent STING1-mediated innate immune response in mouse macrophage RAW264.7 cells (45). Epidermal growth factor receptor (EGFR)-mediated phosphorylation of STING1 on Tyr 245 contributes to STING1 trafficking to Golgi and subsequent activation in RAW264.7 cells, whereas EGFR deficiency leads to autophagosome localization of activated STING1, instead of transport to the Golgi (46). In line with this, the activation of EGFR and ALK receptor tyrosine kinase can mediate STING1 activation in macrophages caused by CDNs (47). STING couples with the downstream kinases of EGFR and PI3K to control the accumulation of F-actin during the activation of B-cell receptor (BCR) (48), highlighting an interplay between STING1 and cytoskeleton in B cells. Overall, these data indicate that a phosphoinositide-dependent cytoskeleton transporting system is highly associated with ER-to-Golgi trafficking of STING1, and this process is fine-tuned through the EGFR signal pathway (**Figure 2**).

Evidence indicates the potential role of TGN in mediating STING1 activation. First, the active form of TBK1 is only localized at the TGN, but not at the rest of the Golgi domains (49). Second, the lipid molecule D-ceramide-C6 disrupts lipid rafts at the Golgi and TGN location of proteins, and inhibits STING1-dependent phosphorylation of TBK1 and IRF3 without affecting the translocation of STING1 to the Golgi (49). Third, sphingomyelin and cholesterol are enriched in the TGN, which contribute to the phosphorylation of STING1 by TBK1 (50). Understanding the active mechanism of STING1 from TGN is therefore important in innate immunity. Whether CGN plays the opposite role in STING1 activation is not fully understood.

STING1 activation is highly dependent on palmitoylation, which is implicated in the clustering of many proteins into cholesterol- and sphingomyelin-enriched lipid rafts and TGN (51). Upon activation, STING1 interacts with several palmitoyl transferases on Golgi, such as zinc finger dhhc-type palmitoyltransferase (ZDHHC) (43, 52) (**Figure 2**). The palmitoylation of two membrane-proximal Cys residues (C88/91) of STING1 is crucial for STING1 activation, even for gain-of-function STING1 mutants in SAVI patients, which makes STING1 constitutively expressed on Golgi (49, 53). Accordingly, nitro fatty acids directly modify STING1 palmitoylation by nitro alkylation, leading to the inhibition of type I IFN production in fibroblasts derived from SAVI patients (54). In addition, 4-hydroxynonenal (4HNE), one of end products of lipid peroxidation, inhibits STING1 translocation to Golgi and activation by the carbonylation of STING1 at C88 in mouse primary peritoneal macrophages (55). This 4HNE-mediated STING1 inhibition is enhanced by the depletion of glutathione peroxidase 4 (GPX4), which is known for specifically catalysing the reduction of lipid peroxides. However, excessive lipid peroxidation can lead to cell death (especially ferroptosis), which may trigger damage-associated molecular pattern (DAMP)-mediated inflammation and immune responses.

In addition, the sulfated glycosaminoglycans (sGAGs) can bind the TM domain of STING1 and promote STING1 clustering in Golgi and subsequent activation in cancer cells (THP1, HeLa, and HT1080 cells) (56) (**Figure 2**). A mutant of STING1 lacking a TM domain and residues C88/91 could form a self-clustered tetrameric structure and effectively trigger the TBK1 and IRF3 activation by cGAMP under physiological conditions (57). Therefore, clustering of STING1 may be a prerequisite for the interaction of STING1 and TBK1, although it is unclear whether this change is different under physiological and pathological conditions.

### Mitochondria

The ER and mitochondria share a close relationship *via* multiple molecular interaction-bridged mitochondria-associated membranes (MAMs), which enable signal messenger movement between the two organelles, regulating mitochondrial fusion and fission, immune response, metabolic homeostasis, and cell death (58). The location of STING1 on mitochondria including MAMs has recently been documented (59). During some RNA virus infections, STING1 serves as an immune adaptor on the outer membrane of mitochondria by interacting with mitochondrial antiviral signaling protein (MAVS). Viral RNA sensor DExD/H-box helicase 58 (DDX58, best known as RIG-I) is recruited to the STING1-MAVS complex on the MAM (4, 60). STING1 then links IRF3 and TBK1 to MAVS, thus transmitting DDX58-MAVS–mediated signals (4, 60). Recently, CGAS-like receptors (CGLRs) were identified as a double-stranded RNA sensors in *Drosophila*, which can produce a novel second messenger, cG[3’-5’]pA[2’-5’]p (3’2’-cGAMP), for activating a STING1-dependent immune response (61). These data imply a crosstalk between CGAS/STING1- and DDX58/MAVS-dependent innate immune response pathways on MAMs (**Figure 1**).

Mitochondria are the central regulator of cell death. STING1 is accumulated in mitochondria in human ferroptotic pancreatic ductal adenocarcinoma cells (62). Although reactive oxygen species (ROS) is closely related to various types of regulated cell death, the specific factors driving STING1 mitochondrial accumulation in ferroptosis are still elusive. Consequently, mitochondrial STING1 promotes ferroptosis sensitivity through binding to mitochondria fusion regulators, namely mitofusins (including mitofusin 1 [MFN1] and mitofusin 2 [MFN2]), to increase mitochondria fusion-mediated ROS production and lipid peroxidation (**Figure 2**). Although these findings establish a direct role of STING1 in mitochondrial dynamics and cell death, the mitochondria fission mediator dynamin 1-like (DNM1L) can function as a negative regulator of STING1-dependent IFNB/IFN-β production (63, 64). The dynamic relationship between mitochondrial DNA damage, cell death, and the STING1 pathway in the control of sterile inflammation and tissue damage needs further investigation.

### Endosomes

Endosomes are essential cellular stations where endocytic and secretory trafficking routes converge. The proteins transiting at endosomes are degraded by lysosomes or recycled to the other organelles (65). STING1 can be translocated to acidified endosomes and finally degraded by lysosomes at the late stage of STING1 activation (66). In contrast, inhibition of this process by the lysosomal inhibitor bafilomycin A1 that targets the fusion step of endosomes and lysosomes could enhance STING1-mediated antitumor immune response (**Figure 2**). Many endosomal interactors with STING1 have been identified at the late activation stage in RAW264.7 cells, providing an integrated mechanism to control STING1 trafficking (43). Unc-93 homolog B1, TLR signaling regulator (UNC93B1) is a chaperone of endosomal toll-like receptors (TLRs) and acts as a repressor of STING1 activation by anchoring STING1 in the endosomes of human and mouse fibroblasts (67). Endosomal TLRs play a role in the activation of B cells in the context of autoimmune diseases (68). STING1-deficient mice had significantly less arthritic joint inflammation, but these mice still produced autoantibodies (69). In contrast, depletion of the chaperone protein UNC93B required for endosomal localization of TLR7 and TLR9 limits autoantibody production in experimental arthritis models (69). Notably, a recent study showed that the STING1 pathway is not essential for the development of experimental SLE in mice, suggesting that other DNA-sensing pathways have alternative roles in mediating autoimmune pathology (70). STING1 can also be exocytosed in CD63^+^ extracellular vesicles, generating an antiviral effect in recipient cells during HSV-1 infection (71–74) (**Figure 2**). The function of extracellular STING1 in the setting of disease remains elusive, but it may represent a mechanism to shape immune response.

### Lysosomes

As mentioned above, after the activation of the CGAS-STING1 pathway, STING1 can be transported to the lysosome through autophagosomes and endosomes for degradation. Lysosomal disorder is associated with increased STING1-mediated immune response, highlighting that lysosomal damage and substance accumulation are the activation signals of the STING1 pathway. Although the mechanism still depends on the context, several proteins, including small GTPase RAB7A (10, 66), autophagy receptor sequestosome 1 (SQSTM1) (75), and lysosomal membrane protein Niemann-Pick type C1 (NPC1) (33), contribute to STING1 translocation to lysosomes for autophagic degradation in various disease conditions (**Figure 2**). This plasticity may explain why the degradation of STING1 needs different stimulation signals and mediators.

The most well-understood autophagy pathway involving ubiquitin is selective autophagy. There is a complex relationship between STING1 and autophagy receptors during stress. SQSTM1 is required for STING1-dependent autophagy to inhibit *M. tuberculosis* infection in macrophages, but SQSTM1 is dispensable for STING1-mediated autophagy in HeLa cancer cells (76). STING1 is degraded in a SQSTM1-dependent manner correlating with its K63-linked ubiquitination in monocyte cells (75, 77). However, in HeLa cells, the autophagy receptor coiled-coil domain containing 50 (CCDC50) binds to and targets K63-polyubiquitinated STING1 for autophagic degradation. Studies reveal that CCDC50 mainly mediates the delivery of STING1 to MAP1LC3-positive autophagosomes, rather than directly promoting STING1 degradation in lysosomes (78). Therefore, each step of selective autophagy degradation of STING1 may require different autophagy receptors to mediate.

The inhibition of STING1 degradation in lysosomes promotes downstream immune responses. A lysosomal protein solute carrier family 15 member 3 (SLC15A3) can interact with STING1 and elevate STING1-dependent type I IFN response, thus protecting against HSV-1 infection in human primary monocytes (79) (**Figure 2**). However, whether and how SLC15A3 mediates the protein stability of STING1 in lysosomes remains obscure.

In addition to immune response regulation, lysosomal STING1 can affect the function of lysosomes in cell death and pH homeostasis (**Figure 2**). In primary monocytes, activated STING1 traffics to the lysosomes, triggering lysosomal membrane permeabilization and subsequently lysosome-dependent cell death (80). Lysosome STING1 also participates in lysosomal rupture, cathepsin B release, and lysosome-dependent cell death in rat neuronal cells after hypoxia ischemia (81). In addition, circulating mitochondrial DNA-mediated robust STING1 activation in macrophages induces STING1 accumulation in lysosomes, which blocks lysosomal acidification and subsequently autophagic clearance of DAMPs in lethal sepsis models (82), suggesting a different anti-autophagy role of lysosomal STING1. Given these contradictory observations, further research is needed to reveal whether the dual role of STING1 in autophagy really depends on its subcellular location. Whether STING1 can act as an autophagy receptor is still unknown.

### The Nucleus

CGAS is present in the nucleus and regulates genomic stability and cell cycle (83–85), raising the possibility that STING1 may have a role in the nucleus. In fact, the nuclear STING1 was originally identified by a proteomics study of the nuclear envelope, and this finding is supported by two subsequent studies, which show a role for nuclear STING1 in promoting chromatin compaction through epigenetic modifications (86–88). STING1 can bind the DNA-dependent protein kinase (DNA-PK) complex in the inner nuclear membrane, which protects breast cancer cells from DNA instability by promoting DDR in a CGAS-independent manner (14). Nuclear STING1 also interacts with various nucleotide-binding proteins, which regulate gene transcription of type I IFNs (89). STING1 redistributes from the nucleus to the ER, increasing both dsDNA- and dsRNA-triggered immune responses. In addition, subcellular localization analysis and the nuclear interactome show that STING1 co-localizes with the lamina, which serves as an anchoring point for chromatin and transcription factors and interacts with transcriptional activators and co-activators (14, 89), indicating that nuclear STING1 may be directly involved in the regulation of gene transcriptional activity (**Figure 2**).

The precise nuclear location and function of STING1 is still controversial. By using structured illumination super-resolution microscopy, STING1 has been found redistributed into different nuclear envelope locations (89). Given that the ER is adjacent to the nuclear envelope, STING1 may diffuse to the contiguous nuclear membrane after its initial insertion into ER membranes (90). Thus, it is not surprising that STING1 localizes on outer and inner nuclear membranes. STING1 can also co-localize with spectrin repeat containing nuclear envelope protein 1 (SYNE1) at the nuclear lamina, and mediates the docking of capsid protein of human herpes viruses to nuclear pore complex proteins (91) (**Figure 2**). These findings provide a framework to explain the import of viral genomes into the nucleus of susceptible cells in the early stages of infection (91). Additional studies are required to fully elucidate the mechanism of ER-located STING1 trafficking into the nucleus and other nuclear functions of STING1, particularly in regulating genome transcription and chromosome stabilization.

## Conclusions and Perspectives

STING1 is a multifaceted protein, probably known best as an adaptor protein of DNA sensor pathways to activate innate immunity. Under normal conditions, most of STING1 is in the ER and acts as a regulator of ER homeostasis. Upon challenge with a range of environmental stresses, STING1 translocates to other organelles, such as Golgi, mitochondria, endosomes, lysosomes, and nuclei, and plays an immune-dependent and -independent role in infection, cell death, and gene expression. Although the subcellular localization sequence of STING1 is unclear, the transport of STING1 in subcellular organelles is regulated by the cytoskeleton, binding proteins, and posttranslational modifications.

This complexity of location and modulation may increase the side effects should STING1 be indiscriminately targeted in diseases. In contrast, using small molecules to target STING1 translocation may be a precision treatment strategy (**Figure 2**). Further studies on the effectors that alter STING1 trafficking and localization may provide novel therapeutic targets for enhancing STING1-dependent immune response or reducing STING1-mediated hyperinflammatory and autoimmune responses. In the meantime, we should uncover the function of cell and tissue type-specific STING1 in health and disease using advanced conditional knockout strategies.

## Author Contributions

RZ wrote the manuscript. RK and DT edited the manuscript. All authors listed have made a substantial, direct, and intellectual contribution to the work, and approved it for publication.

## Funding

This work was supported by grants from the National Institutes of Health of USA (R01CA160417, R01CA211070, and R01GM127791).

## Conflict of Interest

The authors declare that the research was conducted in the absence of any commercial or financial relationships that could be construed as a potential conflict of interest.

## Publisher’s Note

All claims expressed in this article are solely those of the authors and do not necessarily represent those of their affiliated organizations, or those of the publisher, the editors and the reviewers. Any product that may be evaluated in this article, or claim that may be made by its manufacturer, is not guaranteed or endorsed by the publisher.
